# Isolation, Antiradical Activity, and Cytotoxicity of Flavonoids From *Cunila angustifolia*

**DOI:** 10.1002/cbdv.202503539

**Published:** 2026-02-16

**Authors:** Matheus H. O. de Sousa, Marta S. D. Freitas, Karina Cesca, Neusa F. de Moura

**Affiliations:** ^1^ Natural Products Research Group Universidade Federal do Rio Grande Rio Grande Rio Grande do Sul Brazil; ^2^ Universidade Federal de Santa Catarina Florianópolis Santa Catarina Brazil

**Keywords:** acacetin, antiradical, *Cunila angustifolia*, cytotoxicity

## Abstract

*Cunila angustifolia* (“vassourinha do campo”) is a plant species native to southern Brazil that is traditionally consumed as an herbal infusion. In the present study, the hydroethanolic extract of *C. angustifolia* leaves, its solvent‐partitioned fractions, and the flavonoids acacetin and acacetin‐7‐*O*‐rutinoside, reported herein for the first time in this species, were investigated for their antiradical and cytotoxic activities. Among the fractions, the ethyl acetate fraction exhibited the highest total phenolic content (633.7 mg GAE/g) and the strongest radical scavenging activity (EC_50_: 2.0 µg/mL). Comparative evaluation of the isolated flavonoids revealed that acacetin‐7‐*O*‐rutinoside displayed superior antiradical activity relative to its aglycone, suggesting that C‐7 glycosylation may enhance radical scavenging capacity. Cytotoxic assays demonstrated that the crude extract was most active against MCF‐7 breast cancer cells (IC_50_: 37.2 µg/mL), while the chloroform fraction showed selective inhibitory activity against SK‐Mel‐28 melanoma cells (IC_50_: 36.3 µg/mL). In contrast, the isolated flavonoids exhibited weak or selective cytotoxic effects, indicating that the biological activity of the extracts is likely attributable to synergistic interactions among multiple constituents. Overall, these findings expand the phytochemical knowledge of *C. angustifolia* and highlight its potential as a source of bioactive phenolic compounds.

## Graphical abstract

Leaves of *Cunila angustifolia* were extracted and fractionated to isolate flavonoids and terpenoids. Chemical characterization led to the identification of acacetin and acacetin‐7‐*O*‐rutinoside. The ethyl acetate fraction exhibited pronounced antiradical activity, while selective cytotoxic effects were observed against human cancer cell lines, highlighting the biological potential of this species.

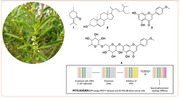

## 1 Introduction

The genus *Cunila* (Lamiaceae) comprises approximately 22 species of aromatic shrubby and herbaceous plants distributed throughout eastern North America and Panama, as well as southwestern South America, including southeastern Brazil, northeastern Argentina, Uruguay, and Paraguay [1]. Owing to their pronounced aromatic profiles, *Cunila* species have attracted considerable attention as sources of essential oils, with numerous studies addressing their chemical composition [2] and associated antibacterial [3], insecticidal [4], and antifungal activities [5]. These investigations have contributed significantly to the understanding of the chemical diversity and ecological roles of volatile metabolites within the genus.

Beyond essential oils, non‐volatile secondary metabolites from *Cunila* species remain comparatively underexplored.

Previous phytochemical studies have reported the occurrence of other classes of natural products, including triterpenes [6] and phenolic compounds, particularly flavonoids [7, 8], alongside enzyme inhibitory effects, such as acetylcholinesterase inhibition [9]. However, systematic investigations correlating the chemical composition of solvent‐partitioned extracts with their biological properties are still scarce.

*Cunila angustifolia* Benth. is a medium‐sized shrubby species native to southern Brazil and extending into the province of Misiones in Argentina [10]. Although it is traditionally used in herbal preparations, phytochemical studies on this species have largely focused on its essential oils. These oils have been classified into three major chemotypes [11]: sabinene, carvacrol oxide, or pulegone‐dominant, depending on geographical site. In addition to their chemical variability, *C. angustifolia* essential oils have been reported to exhibit insecticidal activity [12] and cytotoxic effects against tumor cell lines [13]. In contrast, investigations on the non‐volatile metabolome of this species are limited, with only sporadic reports describing the presence of non‐glycosylated flavonoids oxidized at positions 5, 7, and 4′ or 5, 7, 3′, and 4′ [8].

Given the recognized importance of flavonoids and other phenolic compounds as contributors to chemical biodiversity and mediators of biological activity, a more detailed characterization of the polar constituents of *C. angustifolia* is warranted. In particular, the isolation of individual metabolites, coupled with the evaluation of their biological effects, can provide valuable insights into the contribution of structural features, such as glycosylation, to observed activities, as well as into possible synergistic effects within complex extracts.

In this context, the present study investigates the chemical and biological properties of a hydroethanolic leaf extract of *C. angustifolia*. The work focuses on the isolation and structural elucidation of its major secondary metabolites, followed by a systematic evaluation of the antiradical and cytotoxic activities of the crude extract, its solvent‐partitioned fractions, and the isolated flavonoids. This integrated approach contributes to a deeper understanding of the chemical diversity of *C. angustifolia* and supports its relevance as a source of bioactive phenolic compounds within the Lamiaceae.

## 2 Results and Discussion

### 2.1 Phytochemical Investigation and Structural Characterization

**FIGURE 1:**
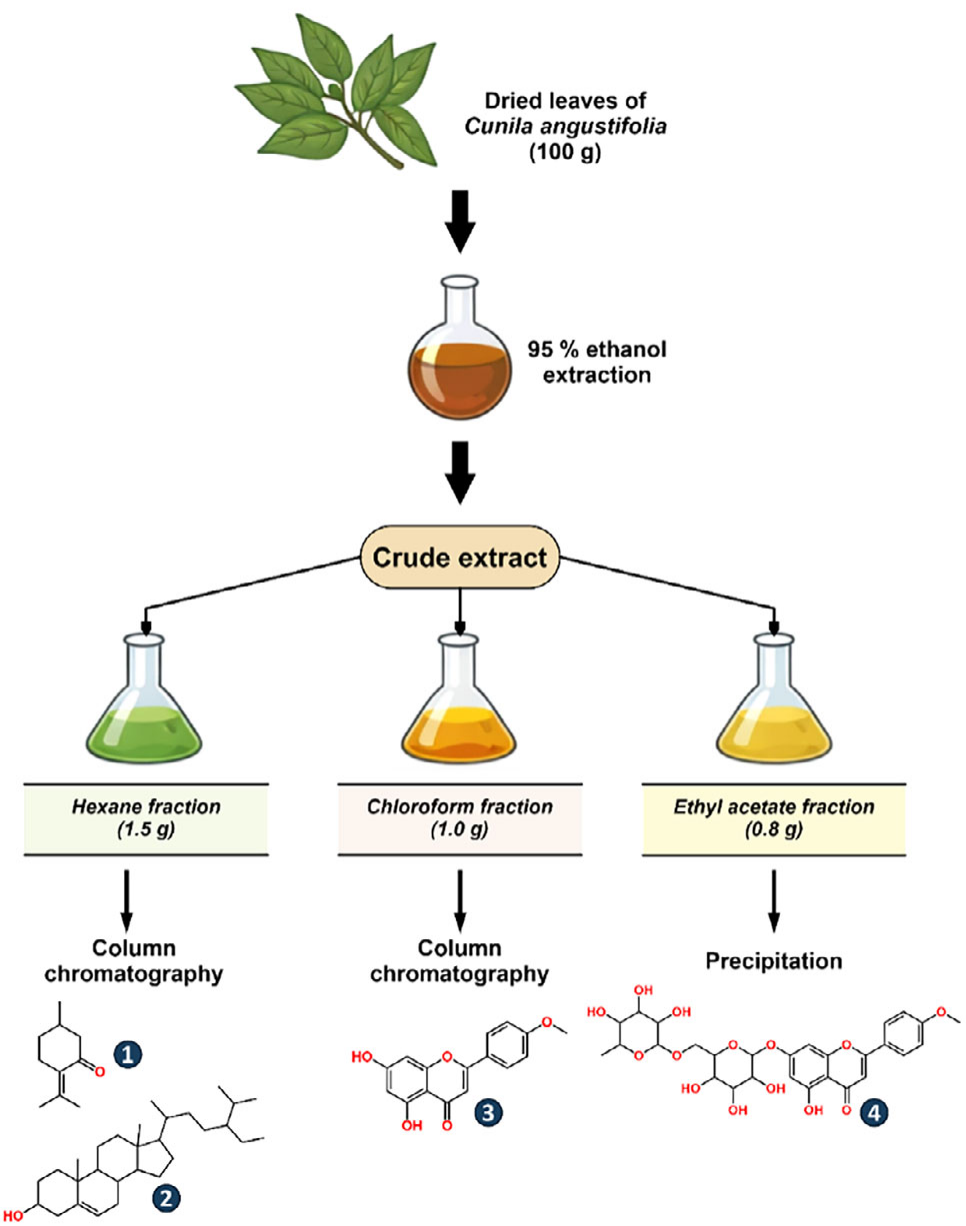
Compounds isolated from the hydroethanolic leaf extract of *C. angustifolia*: pulegone (**1**), *β*‐sitosterol (**2**), acacetin (**3**), and acacetin 7‐*O*‐rutinoside (**4**).

The phytochemical investigation of the hydroethanolic leaf extract of *Cunila angustifolia* led to the isolation of four compounds through successive chromatographic separations. Based on comprehensive spectroscopic analysis and comparison with literature data, the isolated metabolites were identified as pulegone (**1**), *β*‐sitosterol (**2**), acacetin (**3**), and acacetin‐7‐*O*‐rutinoside (**4**) (Figure 1). To the best of our knowledge, this is the first report of these compounds from *C. angustifolia*.

Among the isolated metabolites, pulegone (**1**) is a well‐established monoterpene constituent of *C. angustifolia* essential oils [13], corroborating previous chemotypic studies of the species. Acacetin (ACA), although previously reported from *Cunila lytrifolia* [6], and its glycosylated derivative acacetin‐7‐*O*‐rutinoside (ACG) have not been described for *C. angustifolia* to date. The identification of both aglycone and glycosylated flavonoids highlights the chemical diversity of the polar metabolome of this species and expands current knowledge of secondary metabolites within the genus *Cunila*. The occurrence of *β*‐sitosterol further reflects the widespread distribution of phytosterols among Lamiaceae species.

### 2.2 Total Phenolic Content (TPC) and Antiradical Activity of Extracts and Fractions

Phenolic compounds are widely recognized for their ability to act as radical scavengers, owing to their redox properties and capacity to stabilize free radicals [14]. The TPC of the crude extract and solvent‐partitioned fractions of *C. angustifolia* is summarized in Table 1. Marked differences were observed among samples, reflecting the influence of solvent polarity on phenolic enrichment.

**TABLE 1:** Total phenolic content (TPC) and antiradical activity of the crude extract and solvent‐partitioned fraction of *C. angustifolia* leaves.

Samples	TPC (mg GAE/g)	Antiradical activity (EC_50_ µg/mL)
**CECL**	134.5 ± 15.4	14.6 ± 2.0
**HFCE**	6.4 ± 4.4	>> 250.0
**CFCE**	100.5 ± 13.3	47.1 ± 0.4
**AFCE**	633.7 ± 69.5	2.0 ± 0.1

*Note*: TPC values are expressed as mg gallic acid equivalents per gram of sample (mg GAE/g). Antiradical activity was determined using the DPPH assay and expressed as EC_50_ (µg/mL). Data represent the mean ± SD of three independent experiments.

Abbreviations: AFCE = ethyl acetate fraction, CECL = crude extract, CFCE = chloroform fraction, HFCE = hexane fraction.

The ethyl acetate fraction (AFCE) exhibited the highest phenolic content (633.7 mg GAE/g of fraction), substantially exceeding that of the crude extract and other fractions. This result is consistent with the preferential solubility of medium‐polarity phenolic compounds in ethyl acetate and is in agreement with previous reports on Lamiaceae species [15], where solvent fractionation enhances phenolic concentration as polarity increases.

A clear correlation between TPC and antiradical activity was observed. The AFCE displayed the strongest radical scavenging capacity, with an EC_50_ value of 2.0 µg/mL, surpassing even rutin, used as a reference compound. In contrast, the hexane fraction (HFCE) showed negligible activity, consistent with its low phenolic content. These findings reinforce the central role of phenolic constituents in mediating antiradical activity and underscore the chemical heterogeneity of the extract.

### 2.3 Antiradical Activity of Isolated Flavonoids

The isolated flavonoids ACA and acacetin‐7‐*O*‐rutinoside (ACG) exhibited markedly different antiradical activities (Table 2). Contrary to the general trend reported for flavonoids, where glycosylation often reduces radical scavenging capacity relative to the aglycone [16], ACG demonstrated superior antiradical activity compared to ACA. This observation suggests that glycosylation at the C‐7 position does not necessarily impair radical scavenging efficiency and may, in certain structural contexts, enhance activity. Previous studies have reported that 7‐*O*‐glycosylated flavonoids can retain or even improve antiradical properties depending on the nature of the sugar moiety and experimental conditions such as the solvent utilized or pH [17]. Moreover, the type of sugar moiety can significantly affect activity, with rutinoside residues often conferring greater antiradical capacity than glucoside moieties at the same position.

**TABLE 2:** Antiradical activity of isolated flavonoids from *C. angustifolia* and rutin, determined by DPPH assay.

Samples	Antiradical activity (EC_50_ nmol/mL)
**ACA**	14.6 ± 2.0
**ACG**	>> 250.0
**RUT**	47.1 ± 0.4

*Note*: Results are expressed as EC_50_ values (nmol/L) and represent the mean ± SD of three independent experiments.

Abbreviations: ACA = acacetin, ACG = acacetin‐7‐*O*‐rutinoside, RUT = rutin.

### 2.4 Cytotoxic Activity of Extracts, Fractions, and Isolated Compounds

**FIGURE 2:**
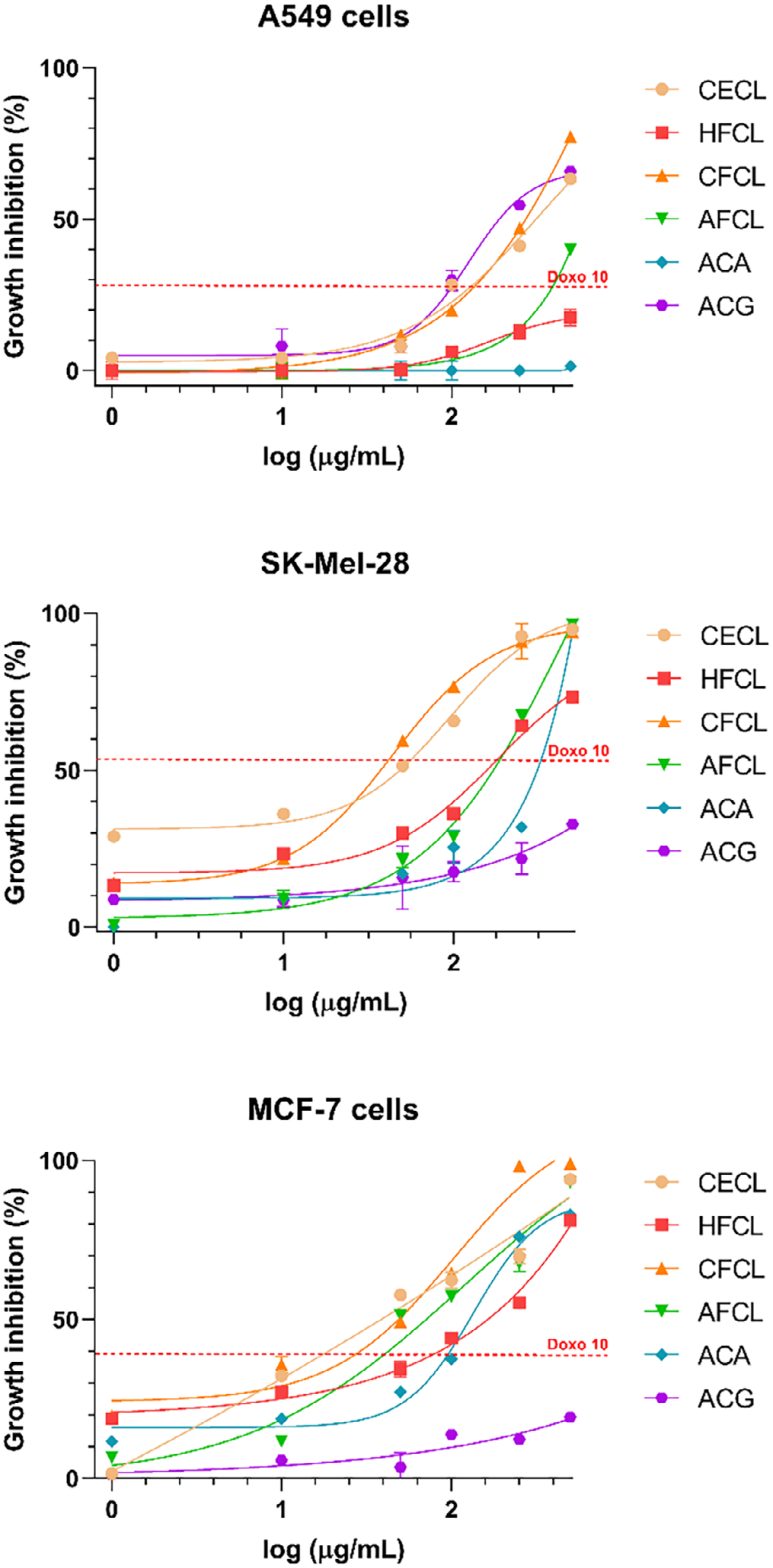
Dose‐response curves showing the cytotoxic activity of the crude extract (CECL), hexane fraction (HFCE), chloroform fraction (CFCE), ethyl acetate fraction (AFCE), and the isolated flavonoids acacetin (ACA) and acacetin‐7‐*O*‐rutinoside (ACG) from *C. angustifolia* after 24 h of treatment. Cytotoxicity was evaluated against A549 (lung carcinoma), SK‐Mel‐28 (melanoma), and MCF‐7 (breast adenocarcinoma) cell lines using the MTS assay. Doxorubicin (10 µg/mL) was plotted as a reference (red dotted line). Data are expressed as mean ± SD from at least three independent experiments performed in quadruplicate.

The cytotoxic activity of the crude extract, fractions, and isolated flavonoids was evaluated against A549 (lung), SK‐Mel‐28 (melanoma), and MCF‐7 (breast) cancer cell lines, as well as non‐cancerous HaCat keratinocytes. The calculated IC_50_ values are presented in Table 3, and dose‐response curves are shown in Figure 2. Neither the crude extract nor the organic fraction exhibited significant cytotoxic effects toward HaCat cells at the concentrations tested.

**TABLE 3:** Cytotoxic activity of the crude extract, solvent‐partitioned fractions, and isolated flavonoids from *Cunila angustifolia* against human cancer cell lines.

	Cytotoxicity (IC_50_ µg/mL)		
Samples	A549	SK‐Mel‐28	MCF‐7
CECL	316.2 ± 1.7	46.8 ± 1.2	37.2 ± 0.7
HFCL	Inactive	151.4 ± 2.5	158.5 ± 2.2
CFCL	269.2 ± 1.4	36.3 ± 0.6	49.0 ± 1.2
AFCL	Inactive	169.8 ± 2.4	69.2 ± 0.8
ACA	Inactive	257.0 ± 1.4	125.9 ± 1.2
ACG	186.2 ± 0.9	Inactive	Inactive
Doxo10	28.8 ± 0.2	53.9 ± 0.9	38.9 ± 0.8

*Note*: Cytotoxicity is expressed as IC_50_ values (µg/mL), calculated from dose‐response curves obtained after 24 h of treatment using MTS assay. Data represent the mean values ± SD derived from at least three independent experiments. Inactive indicates IC_50_ > 500 µg/mL.

Abbreviations: A549, lung carcinoma cells; MCF‐7, breast adenocarcinoma cells; SK‐Mel‐28, melanoma cells.

Overall, the crude extract (CECL) exhibited the highest cytotoxicity across the tested cell lines, particularly against MCF‐7 cells (IC_50_ = 37.2 µg/mL). The chloroform fraction (CFCL) showed selective activity against SK‐Mel‐28 melanoma cells (IC_50_ = 36.3 µg/mL), suggesting enrichment of moderately lipophilic bioactive constituents. In contrast, A549 cells were generally less sensitive to all samples.

Studies with hydroethanolic extracts of *Lamium garnicum* (Lamiaceae) revealed low cytotoxicity against MCF‐7 cells for both the crude extract and its fractions (IC_50_ values in µg/mL: hydroethanolic extract = 124.7, hexane fraction = 293.0, ethyl acetate fraction 101.3) [18]. Similarly, low cytotoxicity has been observed for hydroethanolic extracts from other Lamiaceae species, with IC_50_ values ranging from 134.0 µg/mL in *Matricaria chamomilla* extract to 372.0 µg/mL in *Melissa officinalis* extract [19]. Studies with the A549 cell line indicate that high concentrations of the extracts from the *Salvia* genus are required to induce growth inhibition, with reductions in cell viability observed only above 250 µg/mL, while some tested concentrations remain inactive [20].

The isolated flavonoids exhibited low cytotoxicity against the cell lines tested. Acacetin (ACA) showed moderate activity against SK‐Mel‐28 and MCF‐7 cells, whereas acacetin‐7‐*O*‐rutinoside (ACG) was active only against A549 cells. These differences in activity are likely related to structural variations between ACA and ACG, which may reflect their ability to interact with cellular targets or penetrate the cells. Importantly, the data suggests that the isolated flavonoids are not solely responsible for the low activity observed in the fraction from which they were isolated. For instance, ACA from CFCL and ACG from AFCL exhibited higher IC_50_ values or were inactive compared to their respective fractions.

Acacetin is a widely distributed flavonoid known for its biological properties, including antiproliferative effects against cancer cell lines [21]. Previous studies reported that acacetin inhibited 50% of MCF‐7 cell growth at 26.4 µM [22], exhibited cytotoxicity to A549 cells at concentrations above 5 µM [23], and affected SK‐Mel‐28 cells at 5‐20 µM [24]. Literature also reports IC_50_ values of 103.9 and 157.4 µM for MCF‐7 and A549 cells, respectively [25]. In contrast, our results show that ACA and ACG display relatively low activity against the tested cell lines: ACA was inactive against A549 cells and showed IC_50_ values of 442.9 µM and 904.1 µM for MCF‐7 and SK‐Mel‐28 cells, respectively. ACG was inactive against MCF‐7 and SK‐Mel‐28 cell lines and exhibited an IC_50_ of 314.2 µM for A549 cells.

In general, flavonoids possessing a C2–C3 double bond and a carbonyl group in ring C display the highest antiproliferative activity against cancee cell lines. Substituents at C7, such as in ACG, are associated with a marked reduction in activity [26], which is consistent with the data observed in this study.

## 3 Conclusions

The present study expands the phytochemical knowledge of *Cunila angustifolia* by reporting, for the first time, the occurrence of pulegone, *β*‐sitosterol, acacetin, and acacetin‐7‐*O*‐rutinoside in this species. These findings highlight the chemical diversity of *C. angustifolia*, demonstrating that its secondary metabolome extends beyond volatile constituents to include structurally diverse non‐volatile phenolic compounds.

Among the evaluated samples, the ethyl acetate fraction exhibited the highest phenolic content and the strongest antiradical activity, underscoring the central role of phenolic constituents in radical scavenging. Notably, accetin‐7‐*O*‐rutinoside displayed greater antiradical capacity than its aglycone, emphasizing the influence of glycosylation on biological activity and reinforcing the relevance of structural features in modulating antioxidant properties.

In contrast, cytotoxic evaluation revealed that the crude extract and selected fractions were more active than the isolated flavonoids, indicating that the observed antiproliferative effects are not attributable to individual compounds alone. Rather, these results support the involvement of synergistic or additive interactions among multiple constituents within the extracts, a phenomenon commonly observed in complex natural product matrices.

Overall, this work contributes to a deeper understanding of the chemical biodiversity of *C. angustifolia* extracts and provides a foundation for future studies aimed at elucidating the mechanisms underlying its biological activities. Further investigations focusing on synergistic interactions, bioavailability, and in vivo efficacy will be essential to fully assess the pharmacological potential of this species.

## 4 Experimental Section

### 4.1 General Experimental Procedures

UV‐Vis spectra were recorded using a Shimadzu UV‐Vis 1900 spectrophotometer. Absorbance measurements for cytotoxicity assays were performed on a Spectramax 384 Plus (Molecular Devices). NMR spectra were acquired at 25°C in 5 mm tubes on a Bruker Ascend 400 spectrometer operating at 400 MHz for ^1^H and 100 MHz for ^13^C, using DMSO‐d_6_ or CDCl_3_ as solvent and tetramethylsilane (TMS) as the internal reference. Column chromatography was performed using silica gel 60 (70–230 mesh, Macherey‐Nagel) packed in glass columns with internal diameters ranging from 0.5 to 3.0 cm. Thin‐layer chromatography (TLC) analyses were carried out on pre‐coated aluminum plates (ALUGRAM Xtra Sil G/UV254, Macherey‐Nagel), with visualization under UV light (254 nm) and by spraying with 10% HCl in methanol followed by heating.

### 4.2 Extraction and Isolation

Air‐dried leaves of *C. angustifolia* (100 g) were ground and exhaustively extracted with 95% ethanol at room temperature. The resulting extract was concentrated under reduced pressure to afford the crude extract, which was subsequently partitioned with organic solvents to yield hexane (1.5 g), chloroform (1.0 g), and ethyl acetate (0.8 g) fractions.

The hexane fraction was subjected to column chromatography using hexane‐ethyl acetate mixtures of increasing polarity (0%–100% ethyl acetate), affording 18 fractions. Fractions 1–6, which showed similar TLC profiles, were combined and further purified, leading to the isolation of pulegone (**1**, 4.2 mg) as a colorless oil. Fractions 7–9 yielded crystalline material, which was washed with *n*‐hexane and filtered to afford *β*‐sitosterol (**2**, 3.3 mg) as white needles.

The chloroform fraction was chromatographed using chloroform–ethyl acetate (0%–100% ethyl acetate) followed by ethyl acetate–methanol (0%–100% methanol), yielding 15 fractions. Crystallization observed in fractions 4–6 afforded acacetin (**3**, 225.2 mg) as a yellowish amorphous powder after washing with methanol.

The ethyl acetate fraction, upon dissolution in methanol, yielded a white precipitate that was collected by centrifugation and washed with methanol to afford acacetin‐7‐*O*‐rutinoside (**4**, 325.4 mg) as a white amorphous powder.

### 4.3 Spectroscopic Data

Pulegone (**1**): colorless oil, ^1^H NMR *δ* (400 MHz, CDCl_3_, 7.24), 2.73, 2.70, 1.98, 1.77, 1.01. ^13^C NMR *δ* (100 MHz, CDCl_3_: 77.05): 204.42 (C‐1), 141.99 (C‐2), 131.85 (C‐3), 50.85 (C‐4), 32.80 (C‐5), 31.60 (C‐6), 28.63 (C‐7), 23.03 (C‐8), 22.13 (C‐9), 21.77 (C‐10).

*β*‐sitosterol (**2**): White needles; mp 134°C–136°C. ^1^H NMR *δ* (400 MHz, CDCl_3_, 7.24), 0.94 (*s*, 3H, H‐28), 0.85 (*d*, 3H, *J* = 6.6 Hz, H‐19), 0.77 (*d*, 3H, *J* = 7.0 Hz, H‐24), 0.77 (*d*, 3H, *J* = 7.0 Hz, H‐26), 0.74 (*d*, 3H, *J* = 6.9 Hz, H‐27), 0.61 (*s*, 3H, H‐29), 3.46 (*m*, 1H, H‐3), 5.28 (*d*, 1H, *J* = 5.4 Hz, H‐6). ^13^C NMR *δ* (100 MHz, CDCl_3_, 39.51): 37.5 (C‐1), 31.9 (C‐2), 72.0 (C‐3), 42.5 (C‐4), 141.1 (C‐5), 121.9 (C‐6), 32,1 (C‐7, C‐ 8), 50.3 (C‐9), 36.7 (C‐10), 21.3 (C‐11), 40.0 (C‐12), 42.5 (C‐13), 57.0 (C‐14), 26.2 (C‐15), 28.5 (C‐16), 56.3 (C‐17), 36.4 (C‐18), 19.0 (C‐19), 34.1 (C‐20), 24.5 (C‐21), 46.0 (C‐22), 23.7 (C‐23), 12.2 (C‐24), 29.3 (C‐25), 20.0 (C‐26), 19.2 (C‐27), 19.6 (C‐28), 12.1 (C‐29).

Acacetin (**3**): Yellowish amorphous powder; mp 263°C–266°C. ^1^H NMR *δ* (400 MHz, DMSO‐d_6_, 2.50): 12.9 (1H, *s*, OH–C5), 10.9 (1H, ls, OH–C7), 8.0 (2H, *d*, *J* = 8.9 Hz, H‐2’,6’), 7.0 (2H, *d*, *J* = 8.9 Hz, H‐3’,5’), 6.9 (1H, *s*, H‐3), 6.4 (1H, *d*, *J* = 1.9 Hz, H‐8), 6.1 (1H, *d*, *J* = 1.9 Hz, H‐6), 3.8 (3H, *s*, OCH3). ^13^C NMR *δ* (100 MHz, DMSO‐d_6_, 39.51): 163.7 (C‐2), 104.0 (C‐3), 182.2 (C‐4), 161.9 (C‐5), 99.3 (C‐6), 164.7 (C‐7), 94.5 (C‐8), 157.8 (C‐9), 104.2 (C‐10), 123.3 (C‐1’), 128.8 (C‐2’,6’), 115.0 (C‐3’,5’), 162.8 (C‐4’), 56.0 (OCH3).

Acacetin‐7‐*O*‐rutinoside (**4**): White amorphous powder; mp 253°C–257°C. ^1^H NMR *δ* (400 MHz, DMSO‐d_6_: 2.5): 12.9 (1H, *s*, OH–C5), 8.1 (2H, *d*, *J* = 8.9 Hz, H‐2’,6’), 7.2 (2H, *d*, *J* = 8.9 Hz, H‐3’,5’), 6.9 (1H, *s*, H‐3), 6.8 (1H, *d*, *J* = 2.0 Hz, H‐8), 6.5 (1H, *d*, *J* = 2.0 Hz, H‐6), 5,1 (1H, *d*, *J* = 7.7 Hz, HG‐1), 4.5 (1H, ls, HR‐1), 3.9 (3H, *s*, OCH3), 1.1 (3H, *d*, *J* = 5.7 Hz, HR‐6). ^13^C NMR *δ* (100 MHz, DMSO‐d_6_: 39.51): 163.9 (C‐2), 103.8 (C‐3), 182.0 (C‐4), 161.1 (C‐5), 100.5 (C‐6), 162.9 (C‐7), 94.8 (C‐8), 156.8 (C‐9), 105.4 (C‐10), 122.7 (C‐1’), 128.5 (C‐2’,6’), 114.7 (C‐3’,5’), 162.4 (C‐4’), 99.9 (CR‐1), 70.7 (CR‐2), 70.3 (CR‐3), 72.0 (CR‐4), 68.3 (CR‐5), 17.8 (CR‐6), 99.9 (CG‐1), 73.1 (CG‐2), 76.2 (CG‐3), 69.6 (CG‐4), 75.7 (CG‐5), 66.1 (CG‐6), 55.6 (OCH3).

### 4.4 Determination of TPC

TPC was determined using the Folin‐Ciocalteu method with minor modifications. Samples were solubilized in methanol (1 mg/mL), and 50 µL of each solution was mixed with 250 µL of Folin‐Ciocalteu reagent, followed by 250 µL of saturated sodium carbonate solution (Na_2_CO_3_) and 4.2 mL of distilled water. After vortexing and incubation for 30 min at room temperature, absorbance was measured at 760 nm. Gallic acid was used as a reference standard, and results were expressed as mg gallic acid equivalent per gram of samples (mgGAE/g). All measurements were performed in triplicate.

### 4.5 Antiradical Activity Assay

Antiradical activity was evaluated using the DPPH assay. A methanolic DPPH solution (0.05 mM) was mixed with sample solutions at concentrations ranging from 250 to 1 µg/mL. After incubation for 30 min in the dark, absorbance was measured at 517 nm. The percentage of inhibition was calculated using the formula *I*% = [(*A*_0_ − *A*_s_)/*A*_0_] × 100, where *A*_0_ is the absorbance of control and *A*_s_ is the absorbance of the samples. A graph with percentage inhibition against concentrations was plotted, and from the graph the EC_50_ was calculated. All assays were performed in triplicate.

### 4.6 Cytotoxicity Assay

The cytotoxic activity of the *C. angustifolia* extract, fractions, and isolated flavonoids was evaluated against A549 (lung carcinoma; ATCC CCL‐185), SK‐Mel‐27 (melanoma; ATCC HTB‐72), and MCF‐7 (breast adenocarcinoma; ATCC HTB‐22) human cancer cell lines, as well as the non‐cancerous HaCat keratinocyte cell line (CVCL‐0038), using the MTS assay. Cells were seeded in 96‐well plates at a density of 1 × 10^4^ cells/well and allowed to adhere for 24 h at 37°C in a humidified 5% CO_2_ atmosphere. Cells were then treated with sample concentrations ranging from 500 to 1.0 µg/mL for 24 hrs. Doxorubicin (10 and 50 µg/mL) was used as a positive control, while DMSO served as the negative control.

After treatment, the culture medium was removed, and the cells were washed twice with phosphate‐buffered saline (PBS). A mixture of 100 µL of fresh culture medium and 20 µL of MTS reagent was then added to each well, followed by incubation for 2 h at 37°C. Absorbance was measured at 490 nm using a microplate spectrophotometer, and the optical density values were used to assess the cell viability.

### 4.7 Statistical Analysis

All cytotoxic experiments were performed in quadruplicate and repeated independently. Data were analyzed using GraphPad Prism 8.0 software. Results are expressed as mean ± standard deviation, and IC_50_/EC_50_ values were calculated from nonlinear regression analysis. Statistical significance was assessed using ANOVA followed by Bonferroni's post hoc test, with *p* < 0.05 considered statistically significant (Supporting Information).

## Author Contributions

**Matheus H. O. de Sousa**: conceptualization, methodology, investigation, data curation, formal analysis, writing – original draft preparation, writing – review and editing. **Marta S. D. Freitas**: methodology, investigation, data curation, formal analysis, writing – review and editing. **Karina Cesca**: cytotoxicity assays, data curation, writing – review and editing. **Neusa F. de Moura**: Conceptualization, methodology, writing – review and editing, supervision. All authors have read and agreed to the published version of the manuscript.

## Conflicts of Interest

The authors declare no conflicts of interest.

## Supporting information




**Supporting File 1**: cbdv71001‐sup‐0001‐SuppMat.docx

## Data Availability

The authors have nothing to report.
