# Supplementary material for: Isolation, Antiradical Activity, and Cytotoxicity of Flavonoids From Cunila angustifolia
Source: Chem Biodivers. 2026 Feb 16;23(2):e03539. doi: 10.1002/cbdv.202503539 (PMC12908889; doi:10.1002/cbdv.202503539)
Supplement: Supplementary file 1 — Supporting File 1: cbdv71001‐sup‐0001‐SuppMat.docx [file CBDV-23-e03539-s001.docx]

**Isolation, Antiradical Activity and Cytotoxicity of Flavonoids from *Cunila Angustifolia***

Matheus H. O. de Sousa^a*^; Marta S. D. Freitas^a^; Karina Cesca^b^; Neusa F. de Moura^a^

^a^ Natural Products Research Group, Universidade Federal do Rio Grande. Santo Antônio da Patrulha – RS, Brazil

^b^Universidade Federal de Santa Catarina, Florianópolis – SC, Brazil

*Corresponding author: matheus.furg@gmail.com

**KEYWORDS:** *Cunila angustifolia*, acacetin, antiradical, cytotoxicity.

**ABSTRACT:** *Cunila angustifolia* (“vassourinha do campo”) is a plant species native to southern Brazil that is traditionally consumed as an herbal infusion. In the present study, the hydroethanolic extract of *C. angustifolia* leaves, its solvent partitioned fractions, and the flavonoids acacetin and acacetin-7-*O*-rutinoside, reported herein for the first time in this species, were investigated for their antiradical and cytotoxic activities. Among the fractions, the ethyl acetate fraction exhibited the highest total phenolic content (633.7 mg GAE/g) and the strongest radical scavenging activity (EC_50_: 2.0 μg/mL). Comparative evaluation of the isolated flavonoids revealed that acacetin-7-*O*-rutinoside displayed superior antiradical activity relative to its aglycone, suggesting that C-7 glycosylation may enhance radical scavenging capacity. Cytotoxic assays demonstrated that the crude extract was most active against MCF-7 breast cancer cells (IC_50_: 37.2 μg/mL), while the chloroform fraction showed selective inhibitory activity against SK-Mel-28 melanoma cells (IC_50_: 36.3 μg/mL). In contrast, the isolated flavonoids exhibited weak or selective cytotoxic effects, indicating that the biological activity of the extracts is likely attributable to synergistic interactions among multiple constituents. Overall, these findings expand the phytochemical knowledge of *C. angustifolia* and highlight its potential as a source of bioactive phenolic compounds.

.

1. **NMR spectra of pulegone (1)**


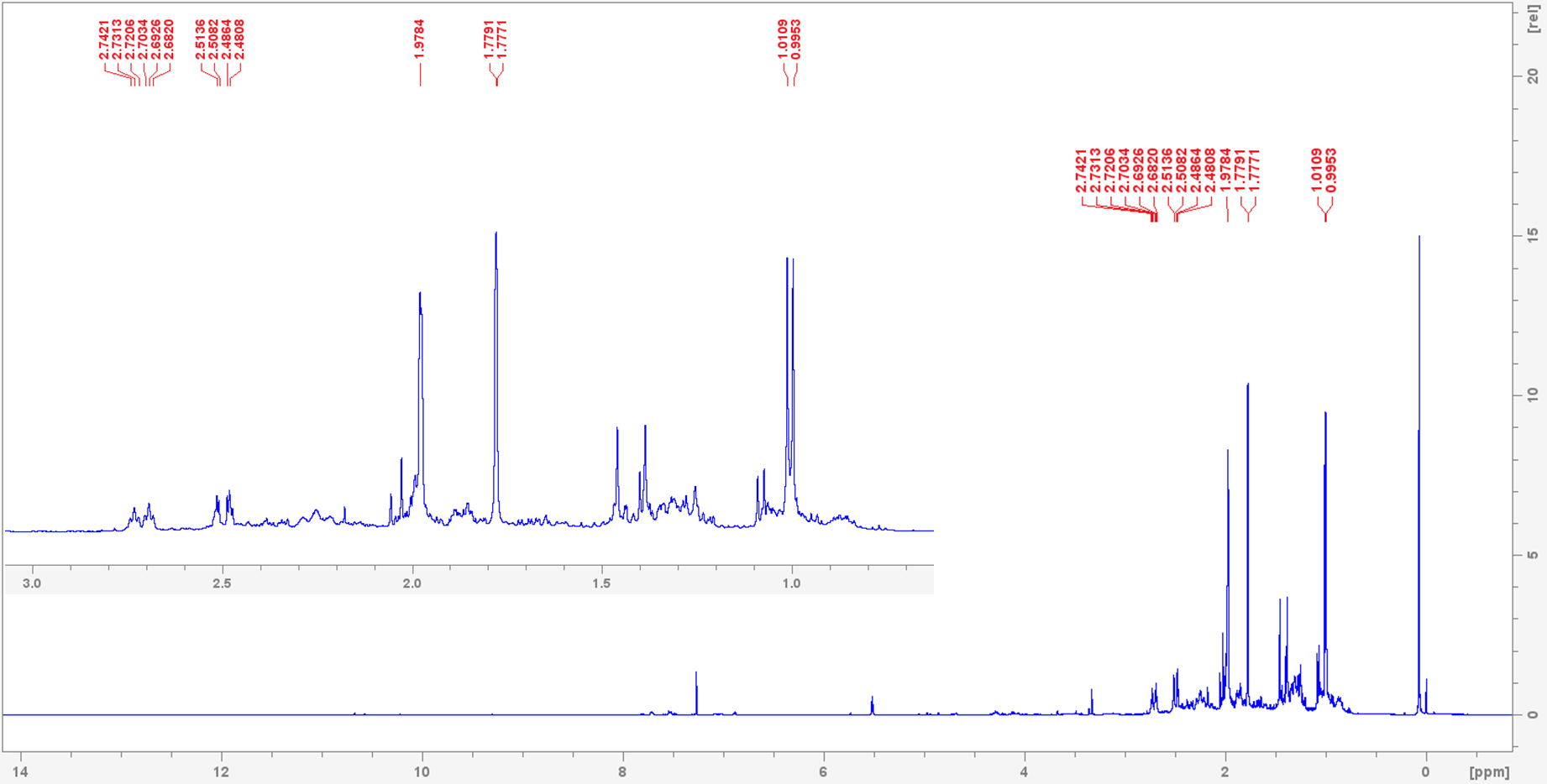


**Figure S1:** ^1^H NMR spectrum of pulegone (**1**) recorded at 400 MHz in CDCl_3_


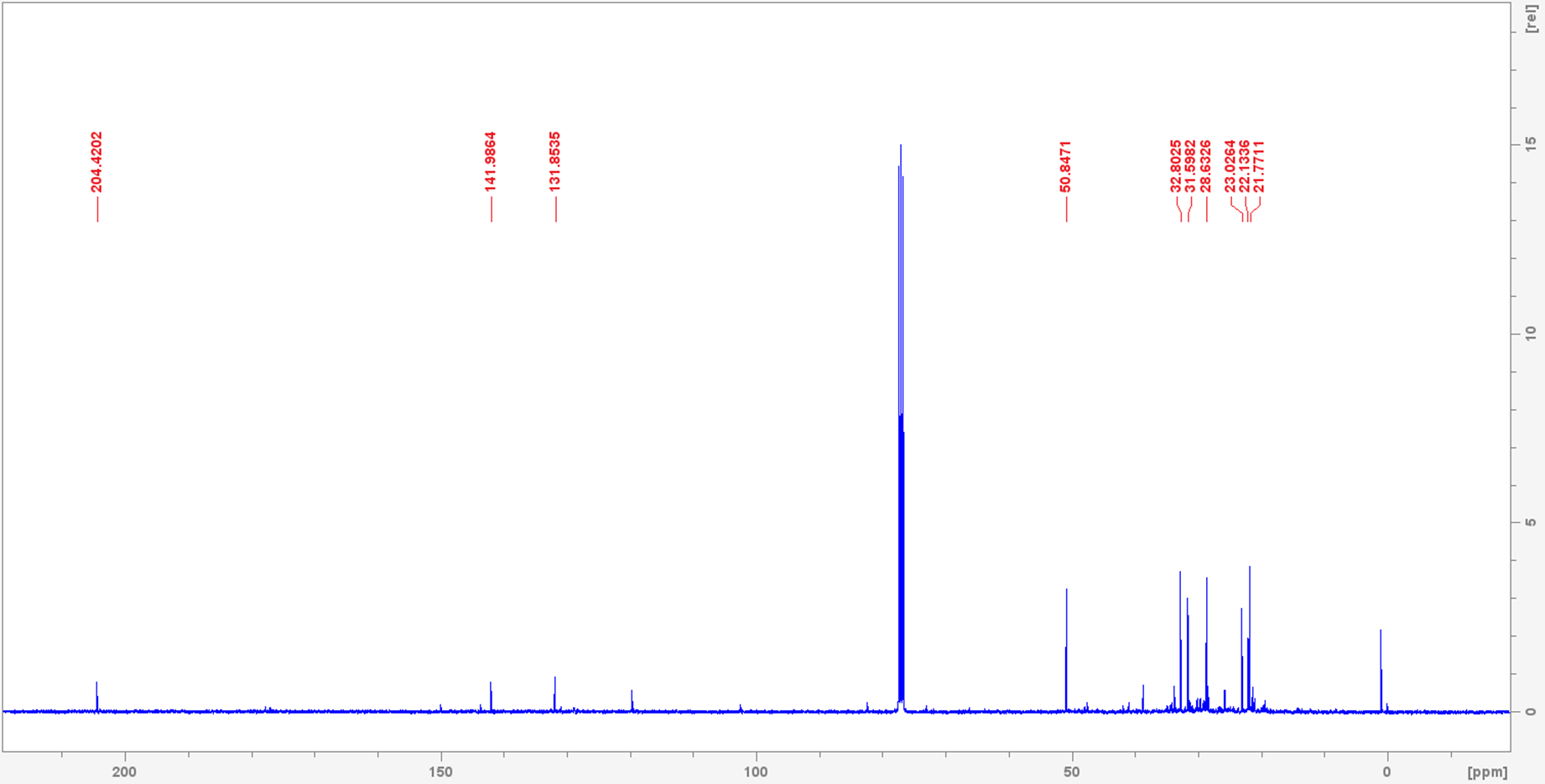


**Figure S2:** ^13^C NMR spectrum of pulegone (**1**) recorded at 100 MHz in CDCl_3_

1. **NMR spectra of *β*-sitosterol (2)**


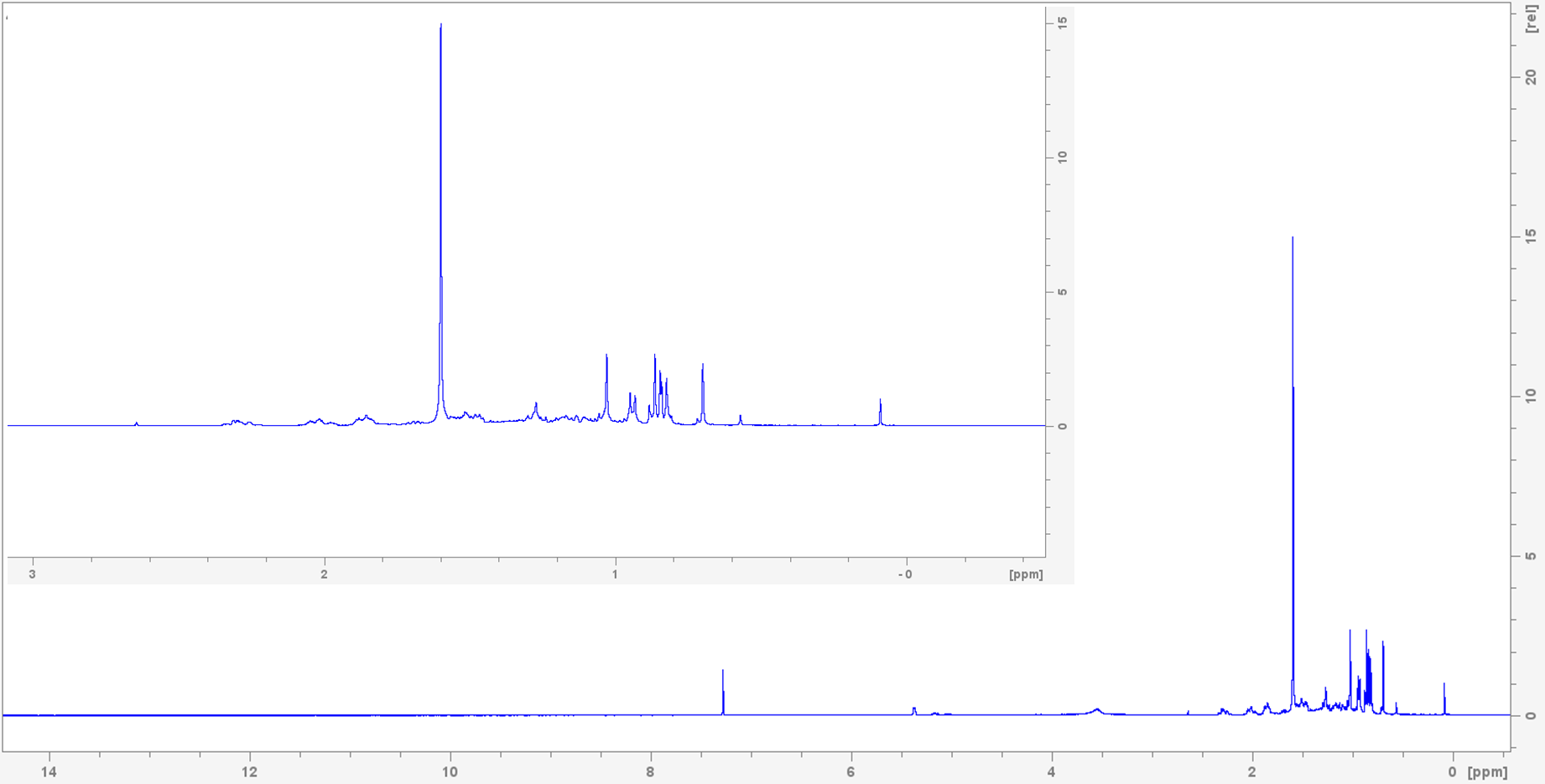


**Figure S3:** ^1^H NMR spectrum of *β*-sitosterol (**2**) recorded at 400 MHz in CDCl_3_


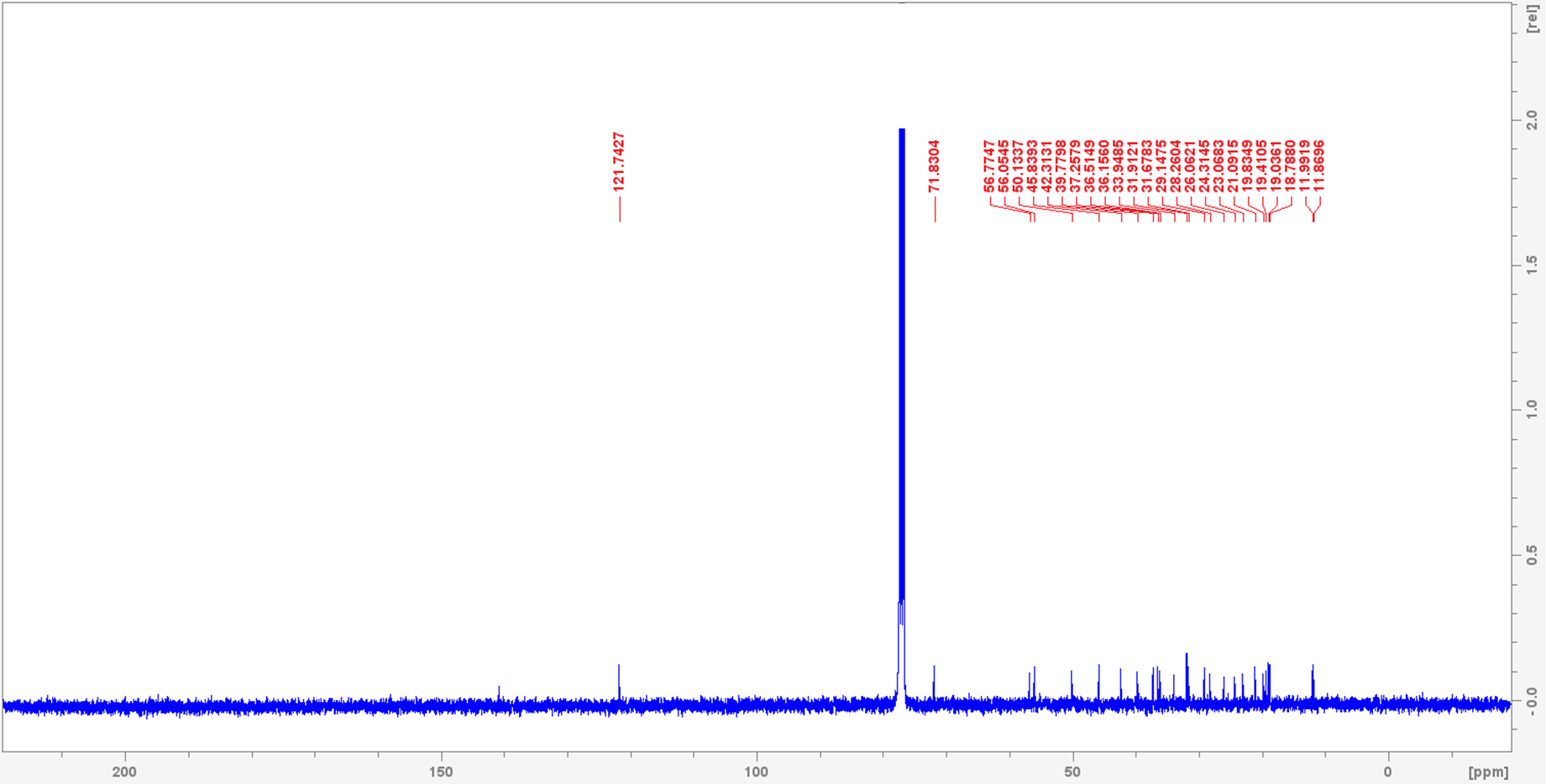


**Figure S4:** ^13^C NMR spectrum of *β*-sitosterol (**2**) recorded at 100 MHz in CDCl_3_

1. **NMR spectra of Acacetin (3)**


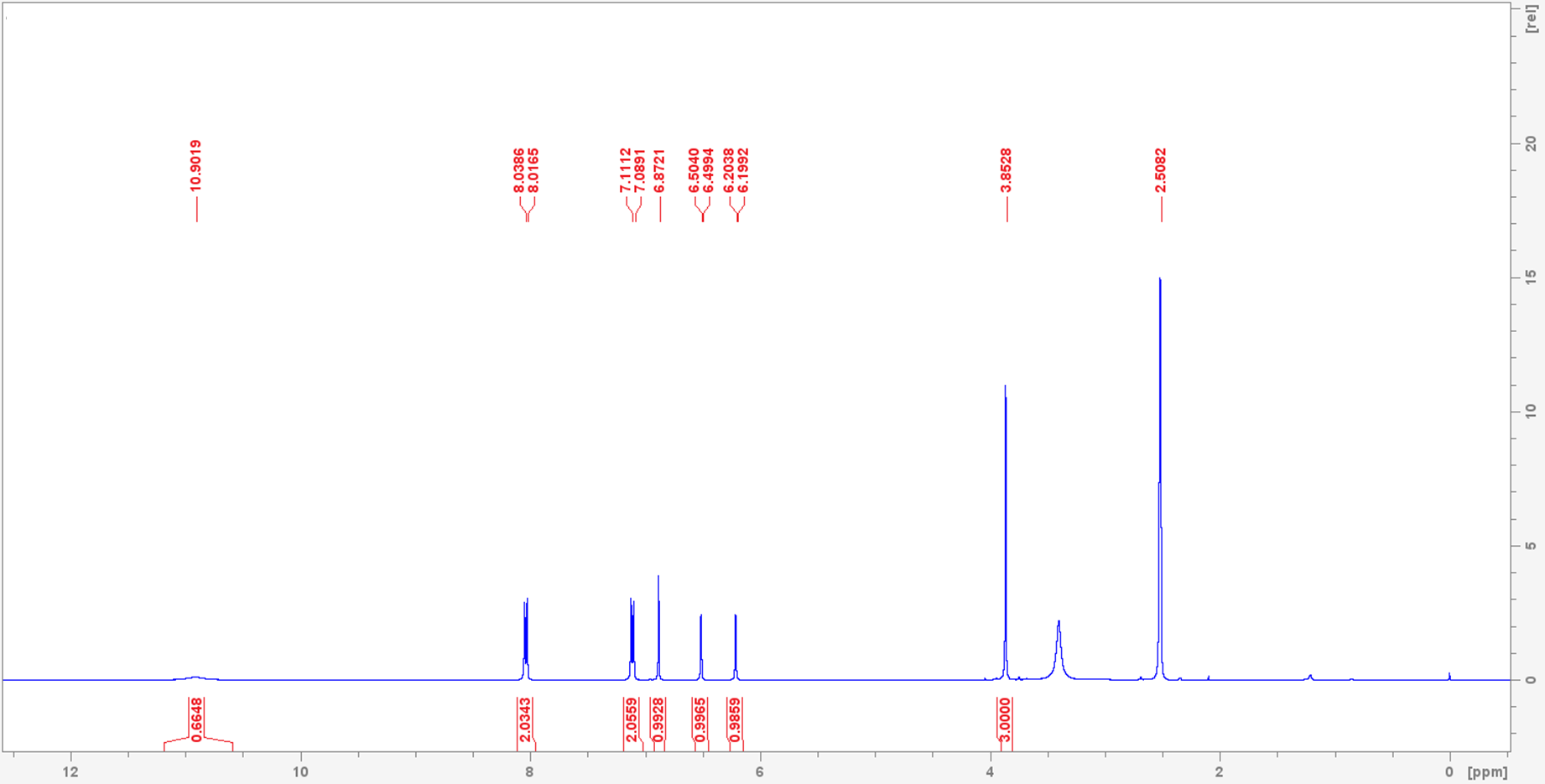


**Figure S5:** ^1^H NMR spectrum of acacetin (**3**) recorded at 400 MHz in DMSO-d_6_


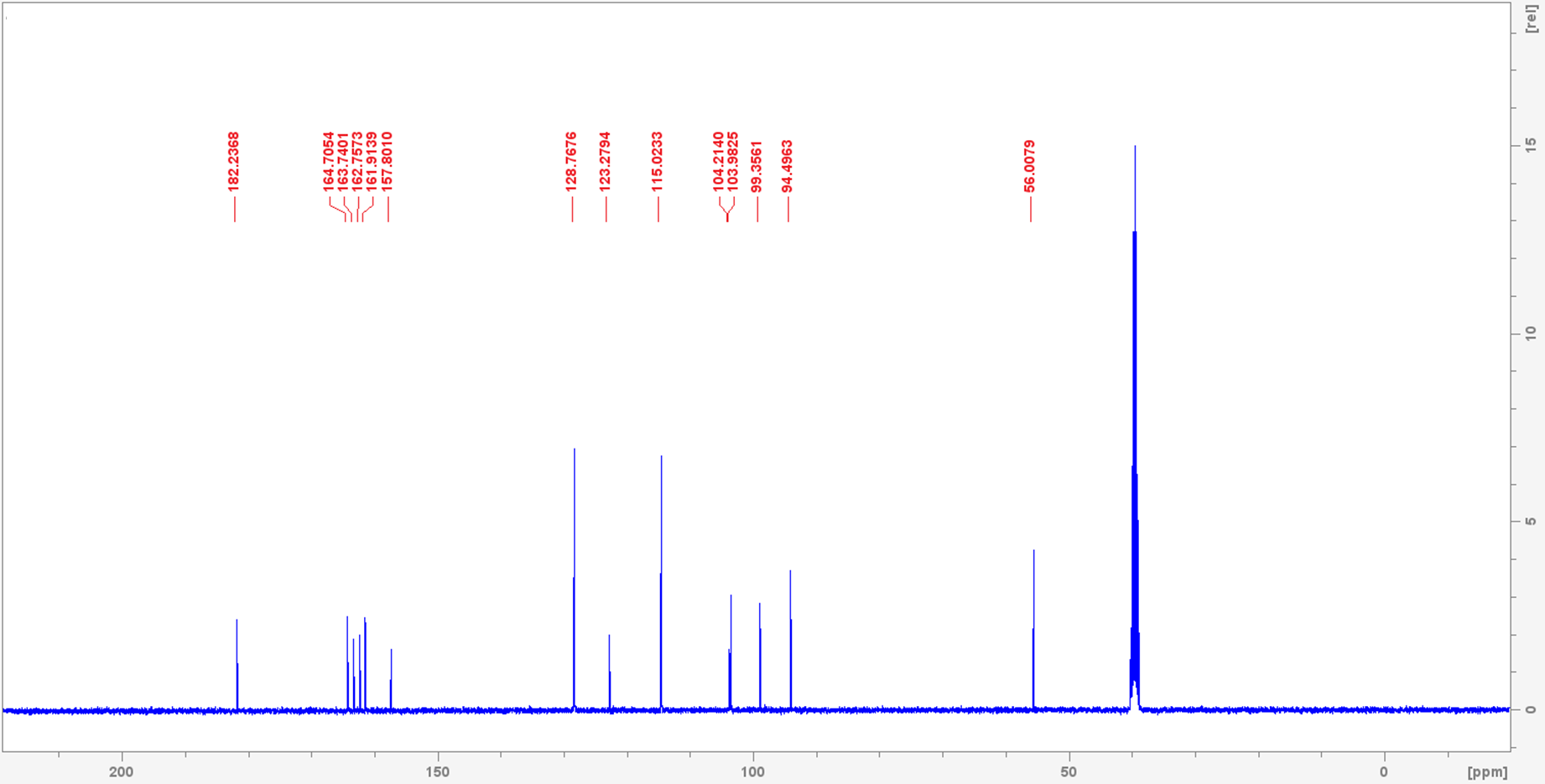


**Figure S6:** ^13^C NMR spectrum of acacetin (**3**) recorded at 100 MHz in DMSO-d_6_

1. **NMR spectra of Acacetin-7-*O*-rutinoside (4)**


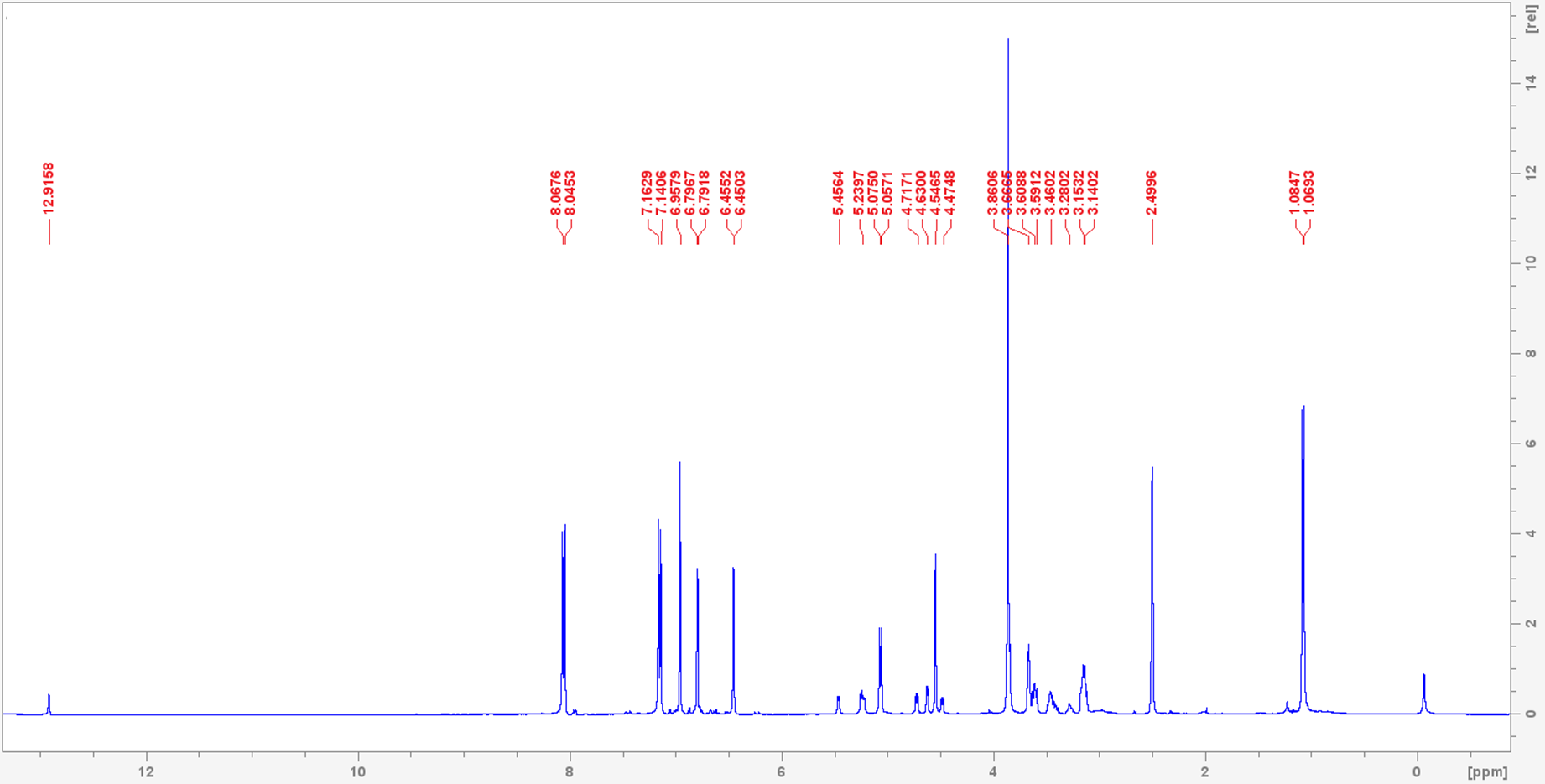


**Figure S7:** ^1^H NMR spectrum of acacetin-7-*O*-rutinoside (**4**) recorded at 400 MHz in DMSO-d_6_


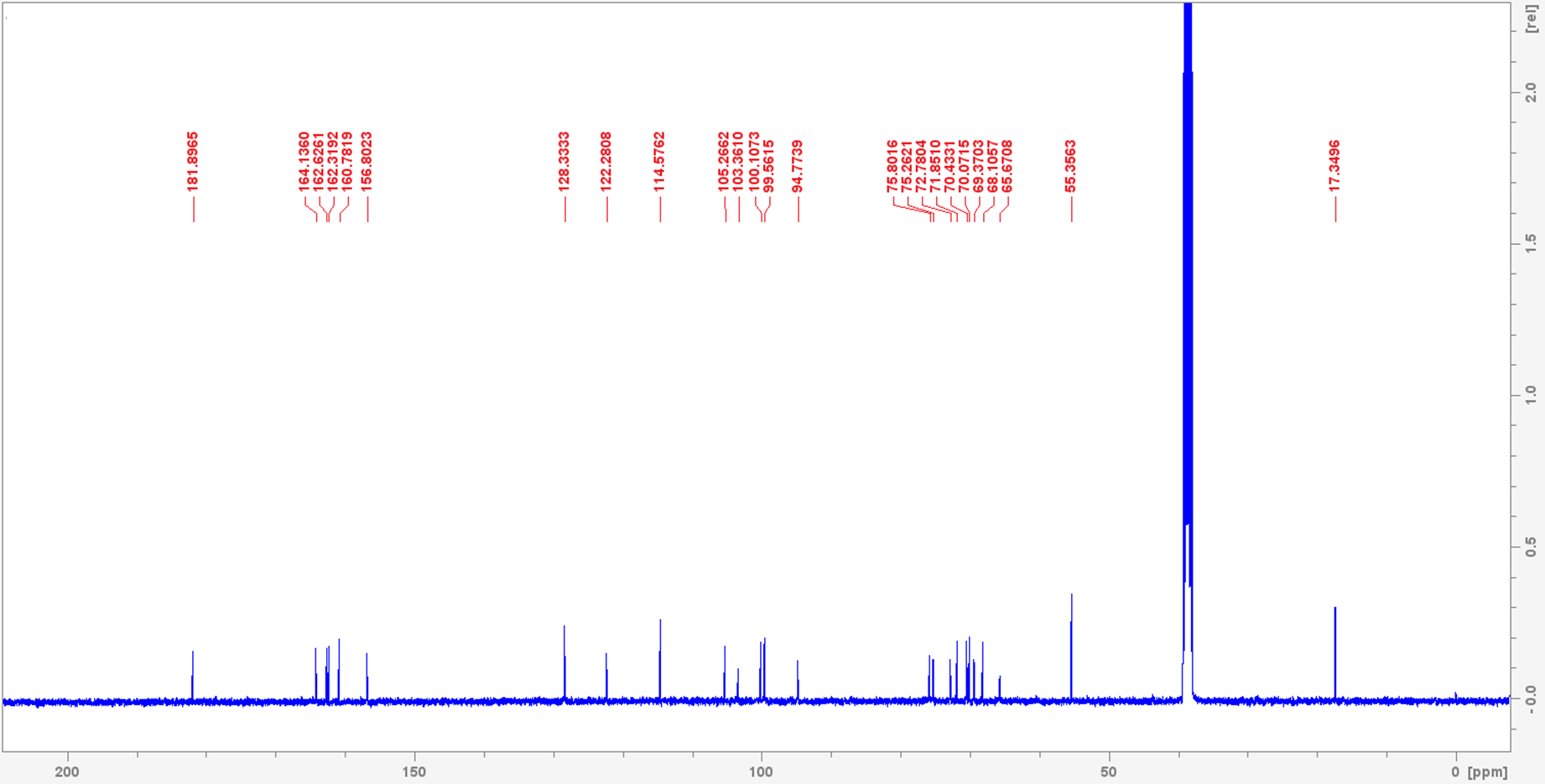


**Figure S8:** ^13^C NMR spectrum of acacetin-7-*O*-rutinoside (**4**) recorded at 100 MHz in DMSO-d_6_

1. **HPLC screening of the crude extracts and isolated flavonoids**

**
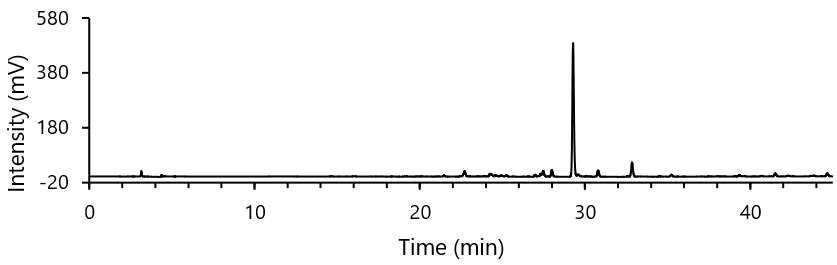
**

**Figure S9:** HPLC chromatogram of the crude hydroethanolic extract of *C. angustifolia* leaves

**
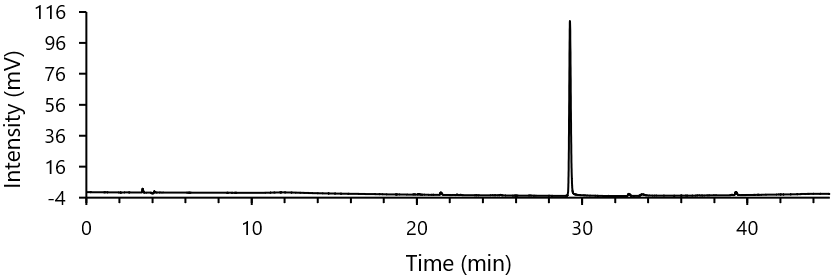
**

**Figure S10:** HPLC chromatogram of acacetin (**3**) isolated from *C. angustifolia* leaves

**
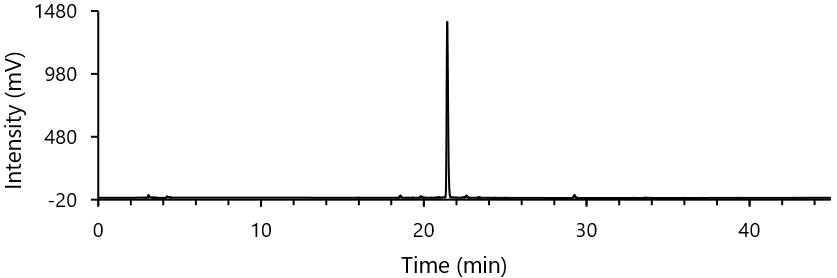
**

**Figure S11:** HPLC chromatogram of acacetin-7-*O*-rutinoside (**4**) isolated from *C. angustifolia* leaves
